# Supplementary material for: Arabidopsis thaliana iron superoxide dismutase FeSOD1 protects ARGONAUTE 1 in a copper-dependent manner
Source: J Exp Bot. 2025 Jun 18;76(18):5465–80. doi: 10.1093/jxb/eraf270 (PMC12596112; doi:10.1093/jxb/eraf270)
Supplement: eraf270_Supplementary_Data [file eraf270_supplementary_data.zip › jexbot315230-file001.pdf]

SUPPLEMENTARY INFORMATION

Supplementary Dataset S1. Nucleotide and amino acid sequences of TST-based inserts.

Supplementary Dataset S2. Proteins identified by AP-MS in *pAGO1:TST-AGO1* and *pAGO1:TST-GFP* transgenic lines grown under Cu deficiency and Cu sufficiency.

Supplementary Dataset S3. Proteins identified by AP-MS in each of the three *pAGO1:TST-AGO1* transgenic lines grown under Cu deficiency versus Cu sufficiency, and not present in *pAGO1:TST-GFP* AP-MS samples for each condition.

Supplementary Dataset S4. Proteins identified by AP-MS in all three *pAGO1:TST-AGO1* transgenic lines grown under Cu deficiency versus Cu sufficiency, and not present in *pAGO1:TST-GFP* AP-MS samples for each condition.

Supplementary Figure S1. Accumulation of miR168 in Arabidopsis Col-0, *fsd1-2* and *ago1-25* seedlings.

Supplementary Figure S2. Quantification of hydrogen peroxide (H_2O_2) content in Arabidopsis Col-0 seedlings under Cu sufficiency and deficiency.

Supplementary Figure S3. AGO1 protein accumulation in Arabidopsis Col-0, *fsd1-2* and *ago1-25* seedlings under oxidative and heavy metal stress.

Supplementary Figure S4. Accumulation of miRNA target mRNAs in different Arabidopsis genotypes under Cu deficiency.

Supplementary Table S1. Name, sequence and use of oligonucleotides used in the present study.

Supplementary Dataset S1. Nucleotide and amino acid sequences of TST-based inserts.

1. Nucleotide sequences

>TST-GFP
AGCGCATGGAGTCATCCTCAATTCGAGAAAGGTGGAGGTTCTGGCGGTGGATCGGGAGGTTCAGCGTGGAGCCAC
CCGCAGTTCGAAAAATCCGGAATGGTGAGCAAGGGCGAGGAGCTGTTCACCGGGGTGGTGCCCATCCTGGTCGAG
CTGGACGGCGACGTAAACGGCCACAAGTTCAGCGTGTCCGGCGAGGGCGAGGGCGATGCCACCTACGGCAAGCTG
ACCCTGAAGTTCATCTGCACCACCGGCAAGCTGCCCGTGCCCTGGCCCACCCTCGTGACCACCCTGACCTACGGC
GTGCAGTGCTTCAGCCGCTACCCCGACCACATGAAGCAGCACGACTTCTTCAAGTCCGCCATGCCCGAAGGCTAC
GTCCAGGAGCGCACCATCTTCTTCAAGGACGACGGCAACTACAAGACCCGCGCCGAGGTGAAGTTCGAGGGCGAC
ACCCTGGTGAACCGCATCGAGCTGAAGGGCATCGACTTCAAGGAGGACGGCAACATCCTGGGGCACAAGCTGGAG
TACAACTACAACAGCCACAACGTCTATATCATGGCCGACAAGCAGAAGAACGGCATCAAGGTGAACTTCAAGATC
CGCCACAACATCGAGGACGGCAGCGTGCAGCTCGCCGACCACTACCAGCAGAACACCCCCATCGGCGACGGCCCC
GTGCTGCTGCCCGACAACCACTACCTGAGCACCCAGTCCGCCCTGAGCAAAGACCCCAACGAGAAGCGCGATCAC
ATGGTCCTGCTGGAGTTCGTGACCGCCGCCGGGATCACTCTCGGCATGGACGAGCTGTACAAGTAA
TST
GFP
ATG Native GFP start codon.
TAA Added STOP codon.

>TST-AGO1
AGCGCATGGAGTCATCCTCAATTCGAGAAAGGTGGAGGTTCTGGCGGTGGATCGGGAGGTTCAGCGTGGAGCCAC
CCGCAGTTCGAAAAATCCGGAGTGAGAAAGAGAAGAACGGATGCTCCATCTGAAGGAGGTGAAGGCTCTGGGTCT
CGTGAAGCTGGTCCAGTCTCAGGTGGTGGACGTGGTTCACAGCGAGGTGGTTTCCAGCAGGGAGGAGGACAACAC
CAAGGTGGAAGGGGTTATACTCCTCAACCTCAACAGGGAGGTCGTGGTGGTCGTGGATATGGGCAACCACCACAA
CAGCAACAACAGTATGGAGGACCACAAGAGTACCAAGGAAGAGGAAGAGGAGGACCTCCTCATCAAGGAGGTCGA
GGAGGGTATGGCGGTGGCCGTGGAGGTGGACCTTCTTCTGGACCACCGCAGAGACAATCAGTTCCCGAGCTGCAT
CAAGCTACCTCACCTACTTATCAAGCGGTGTCTTCTCAGCCTACACTGTCTGAGGTGAGTCCTACCCAGGTACCA
GAACCTACTGTTCTGGCTCAGCAATTTGAACAACTCTCTGTTGAACAAGGAGCTCCCAGTCAGGCAATCCAGCCT
ATACCTTCTTCTAGCAAGGCTTTCAAGTTTCCAATGAGGCCTGGTAAAGGACAGAGTGGAAAGCGTTGCATTGTG
AAGGCTAACCATTTCTTTGCTGAACTGCCTGATAAGGATTTGCACCATTATGATGTTACCATTACTCCGGAAGTT
ACATCAAGGGGTGTCAATCGTGCTGTGATGAAACAACTTGTTGATAATTATCGTGATTCTCACCTTGGAAGTCGT
CTTCCAGCGTATGATGGTCGAAAAAGTCTTTACACTGCTGGTCCACTTCCCTTTAACTCCAAGGAGTTCAGAATC
AATCTTCTTGACGAAGAAGTAGGGGCTGGAGGTCAAAGACGAGAAAGGGAATTTAAAGTTGTGATCAAGCTAGTT
GCACGTGCTGATCTGCATCACCTAGGAATGTTTTTGGAGGGGAAACAATCAGATGCCCCACAGGAAGCTCTGCAG
GTTCTTGACATTGTTCTTCGTGAGCTGCCGACCTCTAGAATCAGGTATATTCCGGTGGGCCGGTCCTTTTATTCC
CCTGATATAGGAAAAAAACAATCATTGGGGGATGGCTTGGAGAGCTGGCGTGGATTCTACCAAAGCATTCGTCCT
ACACAGATGGGCTTATCACTCAATATTGATATGTCATCGACAGCCTTCATAGAGGCAAACCCTGTGATTCAGTTT
GTCTGTGATTTGCTTAACCGGGATATTTCTTCTCGACCTTTATCTGATGCTGATCGTGTTAAGATAAAAAAGGCT
CTTAGAGGTGTCAAAGTTGAAGTGACTCATCGAGGAAACATGCGCCGGAAGTACCGCATTTCCGGTTTGACTGCT
GTGGCCACTCGGGAATTGACATTCCCAGTAGATGAAAGAAATACTCAGAAATCTGTTGTAGAATACTTCCACGAA
ACATATGGTTTTCGCATTCAGCACACTCAACTACCATGCTTGCAAGTTGGGAATTCTAATAGGCCTAATTACTTA
CCAATGGAGGTATGCAAGATTGTTGAAGGCCAGCGGTATTCCAAAAGATTGAATGAGAGACAGATCACTGCTTTG
CTGAAGGTTACCTGTCAGCGCCCGATAGATCGAGAAAAAGATATCTTACAGACGGTGCAACTCAATGATTATGCT
AAAGATAATTATGCTCAAGAGTTTGGCATCAAAATAAGTACTTCTCTGGCTTCTGTTGAGGCTCGTATACTGCCT
CCTCCATGGCTTAAGTACCACGAGTCTGGAAGGGAAGGGACTTGTCTGCCACAAGTTGGTCAATGGAACATGATG
AATAAGAAAATGATCAATGGTGGAACGGTGAATAATTGGATCTGCATCAACTTTTCTAGGCAAGTGCAGGACAAT
CTAGCGCGTACATTTTGTCAGGAACTTGCTCAAATGTGTTACGTATCTGGCATGGCATTTAATCCGGAACCAGTC
CTCCCACCAGTCAGTGCTCGCCCTGAGCAAGTAGAGAAGGTCTTGAAGACTAGATATCATGATGCCACATCAAAA
CTCTCCCAAGGAAAAGAAATTGATCTGCTTATTGTCATTCTGCCCGATAATAATGGATCATTATACGGTGATTTG
AAACGCATATGTGAGACTGAACTCGGCATAGTCTCTCAATGTTGCCTGACAAAGCATGTCTTTAAGATGAGCAAA
CAATACATGGCTAATGTTGCGCTGAAGATTAATGTGAAGGTTGGAGGAAGAAACACAGTGCTTGTTGATGCTCTA
TCTAGGCGGATTCCTCTAGTCAGTGATCGACCCACCATTATATTTGGTGCTGATGTTACCCACCCTCACCCTGGA
GAGGATTCAAGCCCATCTATTGCTGCTGTTGTGGCATCTCAGGATTGGCCTGAAATCACTAAATATGCTGGATTA
GTTTGCGCTCAAGCGCATAGGCAGGAGCTCATTCAGGATCTGTTCAAAGAGTGGAAGGATCCTCAGAAAGGTGTG
GTGACTGGTGGCATGATAAAGGAGTTGCTCATAGCCTTCCGTAGATCAACTGGGCATAAACCACTAAGGATCATC
TTCTACAGGGATGGAGTCAGTGAGGGACAATTTTACCAAGTTTTGCTCTATGAACTTGATGCCATCCGCAAGGCC
TGTGCTTCGCTGGAAGCAGGTTATCAACCACCAGTGACATTTGTGGTGGTGCAGAAGCGTCATCACACGAGGCTG
TTTGCTCAGAACCACAATGATCGCCATTCGGTGGACAGAAGTGGGAATATTTTACCTGGCACTGTTGTGGACTCT

AAAATCTGCCACCCTACAGAGTTTGACTTTTACCTCTGTAGTCATGCTGGTATTCAGGGCACTTCTCGACCTGCT
CATTACCACGTTCTTTGGGATGAGAACAACTTTACTGCAGATGGACTTCAATCTCTGACCAATAACTTATGTTAC
ACGTATGCAAGATGCACACGCTCAGTTTCAATTGTTCCCCCTGCATATTATGCACATCTAGCAGCTTTTAGGGCT
CGATTCTACATGGAGCCAGAGACATCAGACAGTGGCTCAATGGCTAGTGGGAGCATGGCACGTGGAGGTGGAATG
GCTGGTAGAAGCACACGCGGGCCTAATGTCAATGCTGCAGTGAGGCCACTCCCAGCTCTGAAAGAGAATGTGAAG
CGTGTCATGTTCTACTGCTGA
TST
AGO1

2. Amino acid sequences

>TST-GFP
SAWSHPQFEKGGGSGGGSGGSAWSHPQFEKSGMVSKGEELFTGVVPILVELDGDVNGHKFSVSGEGEGDATYGKL
TLKFICTTGKLPVPWPTLVTTLTYGVQCFSRYPDHMKQHDFFKSAMPEGYVQERTIFFKDDGNYKTRAEVKFEGD
TLVNRIELKGIDFKEDGNILGHKLEYNYNSHNVYIMADKQKNGIKVNFKIRHNIEDGSVQLADHYQQNTPIGDGP
VLLPDNHYLSTQSALSKDPNEKRDHMVLLEFVTAAGITLGMDELYK

>TST-AGO1
SAWSHPQFEKGGGSGGGSGGSAWSHPQFEKSGVRKRRTDAPSEGGEGSGSREAGPVSGGGRGSQRGGFQQGGGQH
QGGRGYTPQPQQGGRGGRGYGQPPQQQQQYGGPQEYQGRGRGGPPHQGGRGGYGGGRGGGPSSGPPQRQSVPELH
QATSPTYQAVSSQPTLSEVSPTQVPEPTVLAQQFEQLSVEQGAPSQAIQPIPSSSKAFKFPMRPGKGQSGKRCIV
KANHFFAELPDKDLHHYDVTITPEVTSRGVNRAVMKQLVDNYRDSHLGSRLPAYDGRKSLYTAGPLPFNSKEFRI
NLLDEEVGAGGQRREREFKVVIKLVARADLHHLGMFLEGKQSDAPQEALQVLDIVLRELPTSRIRYIPVGRSFYS
PDIGKKQSLGDGLESWRGFYQSIRPTQMGLSLNIDMSSTAFIEANPVIQFVCDLLNRDISSRPLSDADRVKIKKA
LRGVKVEVTHRGNMRRKYRISGLTAVATRELTFPVDERNTQKSVVEYFHETYGFRIQHTQLPCLQVGNSNRPNYL
PMEVCKIVEGQRYSKRLNERQITALLKVTCQRPIDREKDILQTVQLNDYAKDNYAQEFGIKISTSLASVEARILP
PPWLKYHESGREGTCLPQVGQWNMMNKKMINGGTVNNWICINFSRQVQDNLARTFCQELAQMCYVSGMAFNPEPV
LPPVSARPEQVEKVLKTRYHDATSKLSQGKEIDLLIVILPDNNGSLYGDLKRICETELGIVSQCCLTKHVFKMSK
QYMANVALKINVKVGGRNTVLVDALSRRIPLVSDRPTIIFGADVTHPHPGEDSSPSIAAVVASQDWPEITKYAGL
VCAQAHRQELIQDLFKEWKDPQKGVVTGGMIKELLIAFRRSTGHKPLRIIFYRDGVSEGQFYQVLLYELDAIRKA
CASLEAGYQPPVTFVVVQKRHHTRLFAQNHNDRHSVDRSGNILPGTVVDSKICHPTEFDFYLCSHAGIQGTSRPA
HYHVLWDENNFTADGLQSLTNNLCYTYARCTRSVSIVPPAYYAHLAAFRARFYMEPETSDSGSMASGSMARGGGM
AGRSTRGPNVNAAVRPLPALKENVKRVMFYC

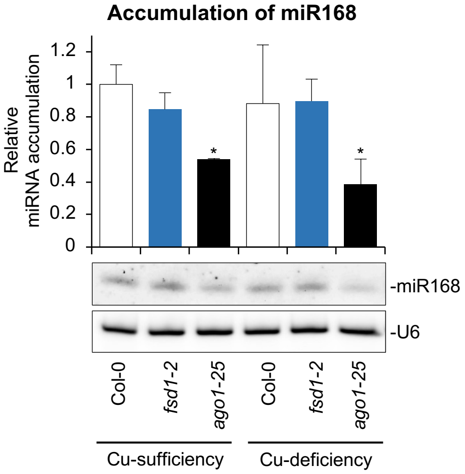

Supplementary Figure S1. Accumulation of miR168 in Arabidopsis Col-0, *fsd1-2* and *ago1-25* seedlings. A, Accumulation of endogenous miR168 miRNA in 10-day-old Col-0, *fsd1-2* and *ago1-25* seedlings, grown under control (Cu sufficiency) and Cu deficiency. The graph at the top shows the mean (n = 3) relative to endogenous miR168 levels + standard deviation (Col-0 = 1.0, under Cu-sufficiency). One representative blot from three biological replicates is shown. Each biological replicate is a pool of at least twenty independent lines that were randomly selected. U6 blot is shown as loading control. Bars with an * are significantly different from that of the Col-0 control sample grown in Cu sufficiency ($P < 0.05$ in all pairwise Student's t-test comparisons).

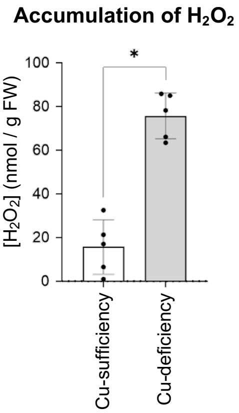

Supplementary Figure S2. Quantification of hydrogen peroxide (H_2O_2) content in Arabidopsis Col-0 seedlings under Cu sufficiency and deficiency. H_2O_2 levels were measured in 10-day-old Col-0 seedlings grown under control (Cu-sufficient) and Cu-deficient conditions. The graph represents means + standard deviation (n = 5 biological replicates, each comprising a pool of >20 randomly selected seedlings, with two technical replicates per sample. The * indicates statistically significant differences ($P < 0.05$, pairwise Student's t-test comparison).

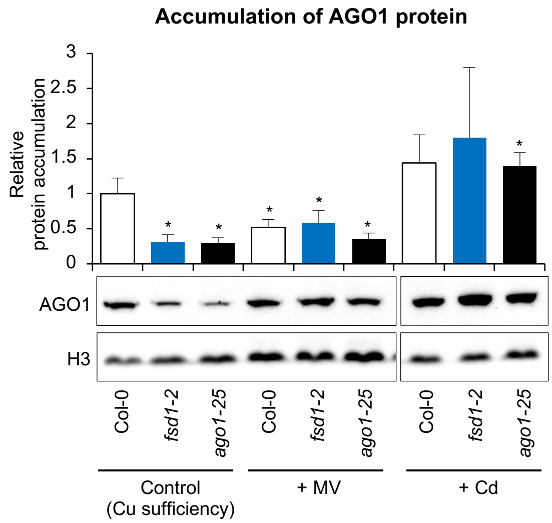

Supplementary Figure S3. AGO1 protein accumulation in Arabidopsis Col-0*, fsd1-2* and *ago1-25* seedlings under oxidative and heavy metal stress. A, Accumulation of endogenous AGO1 protein in 10-day-old Col-0, *fsd1-2* and *ago1-25* seedlings, grown under control (Cu sufficiency) conditions and in media supplemented with methyl viologen (MV, 2 μM) or cadmium (Cd, 30 μM). Graphs at the top show mean AGO1 protein levels + standard deviation (n = 3 biological replicates; Col-0 = 1.0). Each biological replicate is a pool of >20 randomly selected seedlings. One representative blot is shown. HISTONE H3 (H3) serves as loading control of total protein extracts. Bars with an * are significantly different from that of the Col-0 control sample ($P < 0.05$ in all pairwise Student's t-test comparisons).

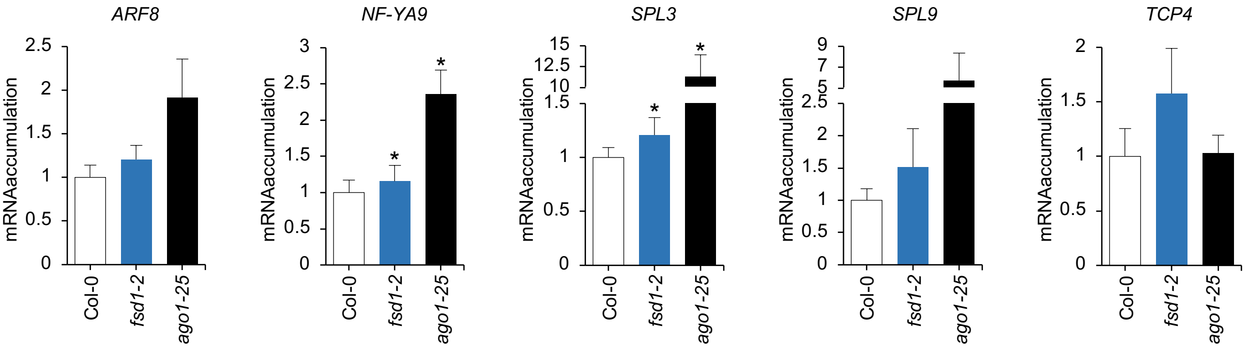

Supplementary Figure S4 . Accumulation of miRNA target mRNAs in different *Arabidopsis thaliana* genotypes under Cu deficiency. The relative expression of *ARF8*, *NF-YA9, SPL3*, *SPL9* and *TCP4* was determined by RT-qPCR in 10-day-old Col-0, *fsd1-2* and *ago1-25* seedlings, grown under Cu deficiency (100 µM BCS). Other details are as in Figure 3A and 3C.

Supplementary Table S1. Name, sequence and use of DNA oligonucleotides used in this study.

Name	Sequence	Type*	Construct/Aim
AC-20	cggccgccccccttcaccatgAGCGCATGGAGTCATCCTC	ssDNA	Generation of *pMDC99-pAGO1-TST-GFP*.
AC-21	tcaacggcgcgcccacccttTTACTTGTACAGCTCGTCCATG	ssDNA	
AC-22	AAGGGTGGGCGCGCCGTT	ssDNA	
AC-23	CATGGTGAAGGGGGGCGGC	ssDNA	
AC-55	AGGGGCCATGCTAATCTTCTC	ssDNA	Probe for U6 detection.
AC-159	AAAAATGGCTGAGGCTGATGA	ssDNA	qPCR amplification of *ACT2* mRNA
AC-160	GAAAAACAGCCCTGGGAGC	ssDNA	
AC-457	T+ACG+CTA+TGT+TGG+ACT+TAG+AA	ssLNA	Probe for tasiR255 detection.
AC-1055	TACCACGAGCTGCGAGAAGAGT	ssDNA	qPCR amplification of *ARF8* mRNA
AC-1056	TTGCGGGAATAACCCACCACTG	ssDNA	
AC-1057	CAAGGTTCAGTTGGTGGAGGA	ssDNA	qPCR amplification of *SPL9* mRNA
AC-1058	TGAAGAAGCTCGCCATGTATTG	ssDNA	
AC-1196	GGGTACTGGAGGACGGTTTG	ssDNA	qPCR amplification of *NF-YA9* mRNA
AC-1197	CGGGGACTGAGTAACATGACC	ssDNA	
AC-1200	CAACCGATACAGGAAACGGAG	ssDNA	qPCR amplification of *TCP4* mRNA
AC-1201	CTGGTATGCGAAAACCCGAAG	ssDNA	
AC-1290	TCGGTGGACAGAAGTGGGAATA	ssDNA	qPCR amplification of *AGO1* mRNA
AC-1291	CCAAAGAACGTGGTAATGAGCAGG	ssDNA	
ARPN-F	TGACTCTCATGGCTGTGTCA	ssDNA	qPCR amplification of *ARPN* mRNA
ARPN-R	CACTACGTTGTGCATCCTCG	ssDNA	
CCS-F	TCTCCACGTCTCTTGGGACTTT	ssDNA	qPCR amplification of *CCS* mRNA
CCS-R	AGCTGAGGCATGGCTCGAT	ssDNA	
CSD1-F	CATCATTGGTCTCCAGGGCT	ssDNA	qPCR amplification of *CSD1* mRNA
CSD1-R	GACCTCCTTATTACATCAAT	ssDNA	
CSD2-F	GTCCTACAACTGTGAAT	ssDNA	qPCR amplification of *CSD2* mRNA
CSD2-R	TCCATGAGGCCCTGGAGT	ssDNA	
D2475	CATGGTGAAGGGGGGCGGC	ssDNA	Generation of *pMDC99-pAGO1-TST-AGO1*
D2476	GTGAGAAAGAGAAGAACGG	ssDNA	
D2477	cggccgccccccttcaccatgAGCGCATGGAGTCATCCTC	ssDNA	
D2478	tccgttcttctctttctcacTCCGGATTTTTCGAACTG	ssDNA	
FSD1-F	ACCGAAGACCAGATTACATA	ssDNA	qPCR amplification of *FSD1* mRNA
FSD1-R	TGGCACTTACAGCTTCCCAA	ssDNA	
GSTU25-F	TGGCAGACGAGGTGATTCTT	ssDNA	qPCR amplification of *GSTU25* mRNA
GSTU25-R	TTGCTAGGCCAAACTTCGTC	ssDNA	
LAC3-F	AACTGCTTTCACCAACCGTC	ssDNA	qPCR amplification of *LAC3* mRNA
LAC3-R	TGGTAGCACGAAGGACATGT	ssDNA	
MSD1-F	GAAGAAGCTAGTTGTTGACAC	ssDNA	qPCR amplification of *MSD1* mRNA
MSD1-R	CCTCGCTTGCATATTTCCAG	ssDNA	
UBQ10-F	TAATCCCTGATGAATAAGTGTTCTAC	ssDNA	qPCR amplification of *ACT2* mRNA
UBQ10-R	AAAACGAAGCGATGATAAAGAAG	ssDNA	

*ssDNA: single-stranded DNA; dsDNA: double-stranded DNA; LNA: locked nucleic acid.
